# Bilateral Symmetric Fracture of the Iliac Wings: An Unusual Situation after a Car Accident

**DOI:** 10.1155/2019/7942904

**Published:** 2019-10-20

**Authors:** Delphine Lambrecht, Jan Van Oost

**Affiliations:** Department of Orthopaedic and Trauma Surgery, AZ Delta Hospital Roeselare, Belgium

## Abstract

**Introduction:**

Pediatric pelvic fractures are uncommon and are usually the result of a high-energy mechanism. Bilateral symmetric fracture of the iliac bone is an extremely uncommon clinical entity that is not yet classified in the current classification systems of pelvic fractures. It mostly occurs among young patients with a history of a seat-belt injury. Patients usually complain of severe hip pain after an accident.

**Case Report:**

A 5-year-old male was transported to our hospital after a car accident. He was complaining of vague pain in the pelvic region after he was exposed to an acceleration-deceleration trauma, seated in a children's car seat. Radiograph of the pelvis revealed a rare image of bilateral symmetric iliac fractures. Iliac bone fracture was suspected, which was also evident on pelvis and hip magnetic resonance imaging. Additional ultrasound of the abdomen was negative. He was hospitalized for observation, and after one day, he could be discharged from the hospital without complications. Policlinic control after three, six, and ten weeks showed favorable clinical and radiographic evolution.

**Conclusion:**

Physicians should be aware of our report, which highlights a patient with the rare clinical condition of a bilateral symmetric fracture of the iliac bone after an acceleration-deceleration trauma. The differential diagnosis of acute hip pain should be considered for young patients. Always keep in mind additional injuries because of the high-energy trauma.

## 1. Introduction

Pediatric pelvic fractures are uncommon with a reported incidence between 0.2% and 2% of all pediatric fractures [1, 2]. Pelvic fractures in children are usually sustained as the result of a high-energy trauma and are commonly associated with concomitant injuries [3, 4]. The classification of pelvic fractures is mostly based on the trauma mechanism [5]. A specific pediatric pelvic classification system of Torode and Zieg describes patterns more commonly seen in the pediatric population [6], although no classification system provides data regarding this specific type of injury.

An extremely rare condition is a bilateral transverse fracture of the pelvis. Documented presentation of this injury in literature is rare. Origin is mostly based on a car accident with seat belt holding the patient on place while the body is bending forward with high force.

We report a similar case of this fracture with no associated lesions.

## 2. Case Report

**Figure 1:**
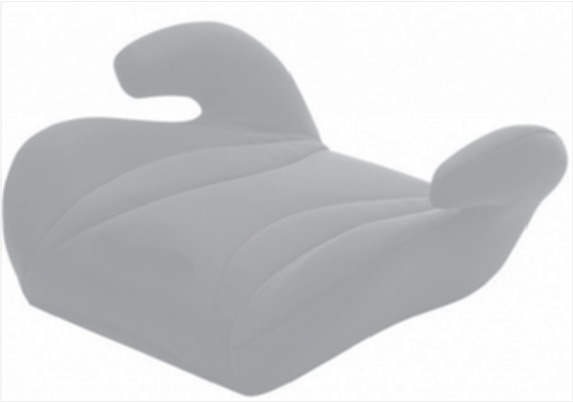
Child car seat.

A 5-year-old boy was involved in a car accident, where the car was catapulted into the ditch. The patient was sitting in the back, on an adapted child car seat (Figure 1), wearing a lap-type seat belt. He was playing computer games while he was sitting bended forward. During the collision, the patient was subjected to an acceleration-deceleration trauma. His head and torso was flexed forward against the front seat, while his pelvic region remained into place by his seat belt.

He never lost consciousness but complained of a headache and pain in his pelvic region. Clinical examination showed a quiet, hemodynamic stable boy with glancing wounds on his head and left hip. He complained of pain in the pelvis and left leg when pushing and mobilization. He had no abdominal discomfort. The patient did not mention any other injuries. There was no neurologic deficit.

**Figure 2:**
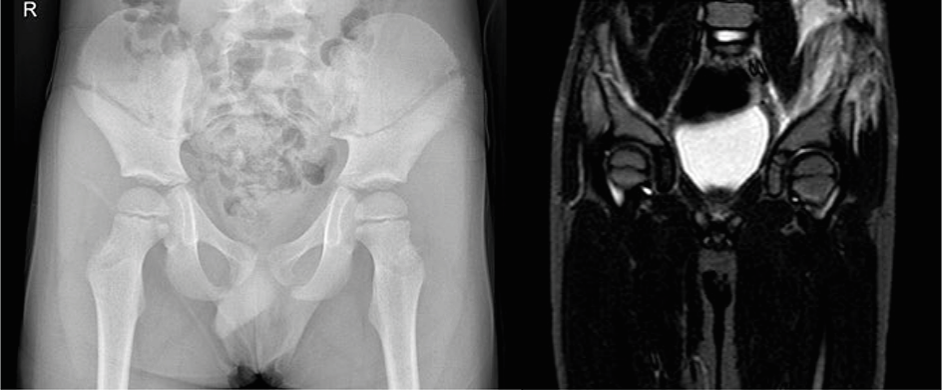
Symmetric bilateral iliac wing fracture with bone and intramuscular edema on additional MRI.

An abdominal ultrasound performed showed no pathologic findings. Radiography showed a bilateral symmetric translucent diagonal line with mild diastasis at the os ileum. This was an atypical image for a fracture. Because there were no obvious clinical symptoms, an additional MRI was executed. Scan confirmed a diagonal fracture of the os ileum with mild diastasis. Additional bone edema and intramuscular edema in the gluteus medius and psoas were visualized (Figure 2).

**Figure 3:**
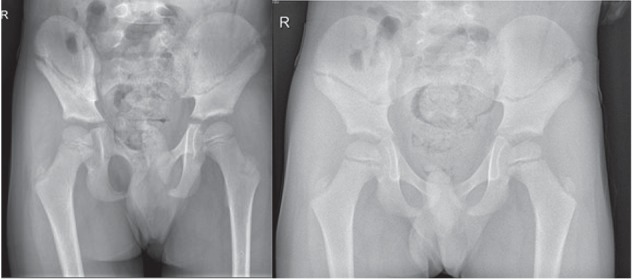
Radiography showed stable condition after three weeks.

Therapy consisted of observation and bed rest. The day after, no important problems were mentioned. The boy could go home with six weeks of nonweight bearing. Three weeks after the accident anteroposterior, inlet and outlet radiographs of the pelvis were made, demonstrating a stable evolution (Figure 3). No big complaints were mentioned.

**Figure 4:**
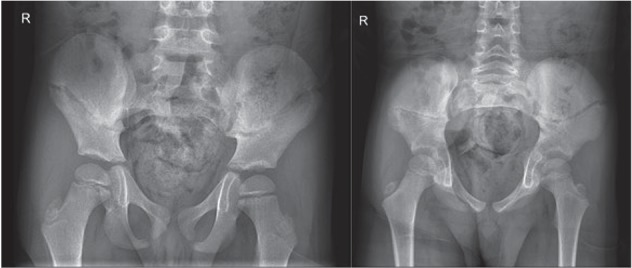
Radiography with formation of callus visible after six weeks.

Six weeks later, radiographies showed endostal callus formation (Figure 4). Mobilization was allowed. He had no pain. Sporting was still prohibited during one month. Last control four weeks later showed a happy boy without complaints. For reasons of radioprotection, no control X-ray was made.

## 3. Discussion

Pediatric pelvic fractures in general are relatively rare. Mostly, children with open triradiate cartilage have different and less severe fracture patterns than those of adults. Explanation can be found in the fact that the cartilage of the open iliac wing is weaker than the elastic pelvic ligaments, resulting in bony failure before pelvic ring disruption. Because of the greater plasticity of the pelvic bones and the increased elasticity and flexibility of the joints in the immature population, more energy is absorbed before a fracture occurs [7].

Therefore, these injuries are always a marker of high-energy trauma, and there must be high suspicion of associated injuries as abdominal and head trauma [8].

Anatomical differences between adults and children cause also different management strategies in pediatric population [8, 9]. Although very few pediatric pelvic fractures will ultimately need surgical treatment, patients with these injuries must be followed over time to confirm proper healing, ensure normal pelvic growth, and address any potential complications [10, 11]. Recovery often depends on associated injuries [12].

Considering classification systems, most of these classifications are based on the injury mechanism linked to the acting forces causing the fracture, stability, and pathoanatomy.

Two classification systems for pelvic fractures are most used. (a) Tile classification accords to fracture pattern and stability. An iliac wing fracture is classified as type A1 (stable fracture not involving the ring, caused by a direct blow) [5]. (b) The Young-Burgess classification accords to the direction of impact force (vertical shear, lateral or anteroposterior compression, and combined) [13]. In our case, anteroposterior forces had an impact, but the case cannot be classified in this system.

**Figure 5:**
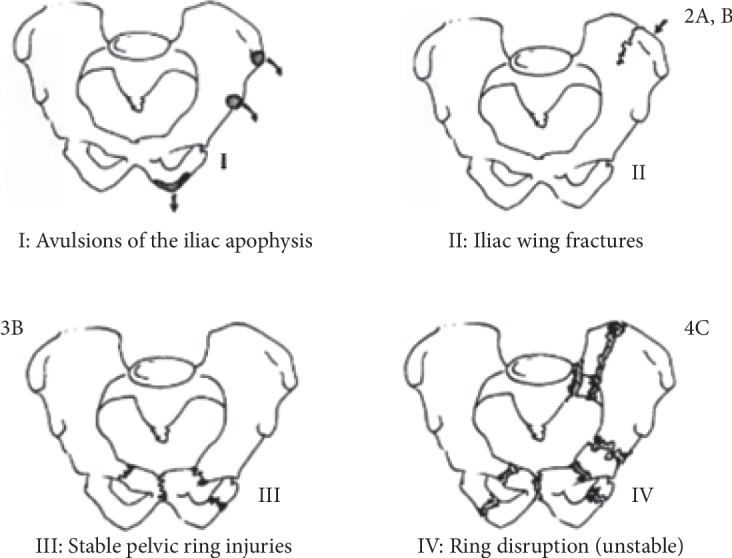
Torode and Zieg classification [6].

The Torode and Zieg classification (Figure 5) is the most popular system used in classifying pediatric pelvic fractures. There is no ideal system to address the wide skeletal maturity variation of pediatric fracture patterns, but it does include the fracture patterns most commonly seen in the pediatric population [7]. Iliac wing fracture is classified as type 2. This fracture type results from a direct force against the pelvis. Most patients were hospitalized for observation of associated injuries. Additional lesions are less frequent compared to type 3 and 4. This can be explained by the interference of a lesser amount of energy trauma. Good results were noted with a short period of bed rest until stabilization. Transient chance in the growth of the iliac apophysis was noted in few patients. Making inlet and outlet views, additional to classic anteroposterior views, provides significant additional information regarding the configuration of the bony pelvis [6].

A symmetric bilateral transverse fracture of the ossi ileum is extremely rare. A thorough review of the literature describes only three reports of this type of fracture [14–16]. Treatment was always conservative with observation. No associated injuries or notable complications were mentioned. Patient in our case recovered fast. Long-term follow-up in these cases is not described.

A good classification system of this bilateral, symmetric fracture does not exist. In our case, injury was caused by an acceleration-deceleration trauma causing a flexion-distraction force. The patient was projected forward sitting in his seat with holding him on place by his seat belt. Most probably, the belly belt caused this type of horizontal splitting fracture.

The mechanism of injury is comparable in other cases [14–16] which causes the same type of injury. In Ofiram et al. [14], the patient was not wearing a belt, but the same acting forces could have interacted.

## 4. Conclusion

In conclusion, this case report illustrates the rare condition of a bilateral symmetric fracture of the iliac bone after a car accident with acceleration-deceleration forces on the pelvic of a child in an adapted car seat. Only three similar cases were found in literature. It is an important trauma in which we advise to be aware of additional injuries.

Although it is very uncommon, we want to consider the need for a different classification system in this type of pediatric pelvic fractures.
